# Development and Application of Whole-Cell Biosensors for the Detection of Gallic Acid

**DOI:** 10.1021/acssynbio.2c00537

**Published:** 2023-02-01

**Authors:** Ingrida Kutraite, Naglis Malys

**Affiliations:** † Bioprocess Research Centre, Faculty of Chemical Technology, Kaunas University of Technology, Radvilėnų Street 19, LT-50254Kaunas, Lithuania; ‡ Department of Organic Chemistry, Faculty of Chemical Technology, Kaunas University of Technology, Radvilėnų Street 19, LT-50254Kaunas, Lithuania

**Keywords:** gallic acid, inducible gene expression system, transcription factor, promoter, biosensor

## Abstract

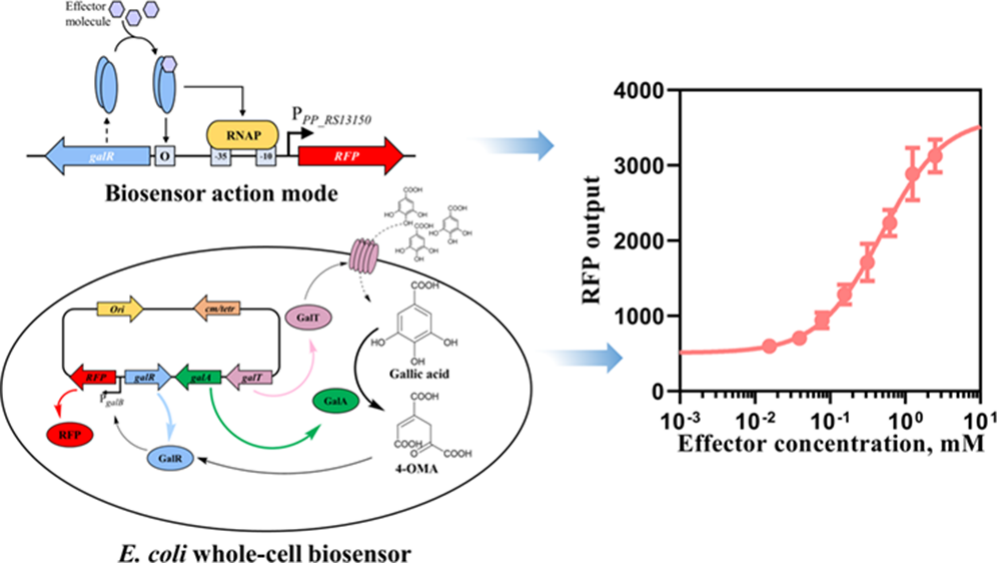

Gallic acid is a prevalent secondary plant metabolite distinguished as one of the most effective free-radical scavengers among phenolic acids. This compound is also known for its cytotoxic, anti-inflammatory, and antimicrobial activities. Bulk quantities of gallic acid are conventionally produced by acid hydrolysis of tannins, a costly and environmentally hazardous process. With the aim to develop more sustainable approaches, microbial bioproduction strategies have been attempted recently. To advance synthetic biology and metabolic engineering of microorganisms for gallic acid production, we characterize here a transcription factor-based inducible system *Pp*GalR/P_*PP_RS13150*_ that responds to the extracellular gallic acid in a dose-dependent manner in *Pseudomonas putida* KT2440. Surprisingly, this compound does not mediate induction when *Pp*GalR/P_*PP_RS13150*_ is used in non-native host background. We show that the activation of the inducible system requires gallate dioxygenase activity encoded by *galA* gene. The 4-oxalomesaconic acid, an intermediate of gallic acid-metabolism, is identified as the effector molecule that interacts with the transcription factor GalR mediating activation of gene expression. Introduction of *galA* gene along *galR* enables development of biosensors suitable for detection and monitoring of gallic acid extracellularly using non-native hosts such as *E. coli* and *C. necator*. Moreover, the *P. putida*-based biosensor’s applicability is demonstrated by detecting and measuring gallic acid in extracts of *Camellia sinensis* leaves. This study reports the strategy, which can be applied for developing gallic acid biosensors using bacterial species outside *Pseudomonas* genus.

## Introduction

Gallic acid, also known as 3,4,5-trihydroxybenzoic acid, is a secondary plant metabolite occurring as a free phenolic acid or as part of polyphenols catechins and tannins. Due to three hydroxyl groups present in the structure, it is known as an outstanding antioxidant among phenolic compounds, which also exhibits a range of other bioactivities including anti-inflammatory, antimicrobial, and even anticancer.^1^ Notably, the anticancer activity of gallic acid has been shown to be destructive to the malignant cells with only negligible effect on the normal tissue, suggesting a wider scope for application in cancer treatment.^2^

Gallic acid and its esters are widely applied in the pharmaceutical, food, dye, paper, cosmetic, and chemical industries.^3,4^ The annual demand for gallic acid has been valued at 74 million USD in 2020 and is expected to reach 125.6 million USD by 2026.^5^ This acceleration is driven by an increased need for this compound from various end-use industries and rising health-consciousness in society.^4^ In addition, although gallic acid can be utilized directly in diverse applications, its value can be enhanced by generating a variety of derivatives, such as methyl, ethyl, propyl, butyl, and octyl gallates, which are being used as antioxidants, food additives, or therapeutic agents.^6,7^ Only a limited amount of gallic acid is obtained from natural sources. It is mostly derived chemically by the acid hydrolysis of tannic acid, the process that is environmentally hazardous and economically unsustainable due to a particularly low purity and yield.^2^ An efficient release of gallic acid from tannins can be achieved using tannases, tannin acylhydrolases, produced by microorganisms.^8,9^ An enzymatic tannase-based approach has been recently utilized for gallic acid production from tannin-rich biowastes using solid-state fermentation.^10,11^

A significant effort has been directed toward the engineering of microbial strains to develop and improve the bioproduction of gallic acid.^12−14^ A structure-based rational engineering of PobA of *Pseudomonas aeruginosa* has allowed generating a *p*-hydroxybenzoate hydroxylase variant with a higher activity toward 3,4-dihydroxybenzoic acid, which has been used to build an artificial biosynthetic pathway for gallic acid biosynthesis in *Escherichia coli*.^15−17^ Engineered biosynthetic pathways have also been developed for gallic acid production from lignin-derived substrates such as syringate, ferulate, and *p*-coumarate in *E. coli*(12,18) and coniferyl alcohol, *p*-coumarate, ferulate, *p*-hydroxybenzoate, protocatechuate, sinapate, syringaldehyde, syringate, and vanillate in *Rhodococcus opacus*.^19^ Notably, *E. coli* has been engineered recently to produce gallic acid from terephthalic acid, a monomeric constituent of polyester that is a major environmental pollutant.^20^

Microbial strain engineering and synthetic biology require robust tools that can be used for fine-tuning of gene expression or monitoring intracellular and extracellular metabolite changes. Although several well-characterized inducible promoters are available for controlling gene expression in diverse bacterial species,^21−24^ ultraviolet–visible spectroscopy or mass spectrometry-based analytical technologies can be applied for measuring metabolite concentrations,^25,26,3^ inducible gene expression systems on their own or in combination with fluorescence, or luminescence reporters can serve both purposes.^27−30^ An inducible gene expression system that responds to exogenous gallic acid has been identified in *P. putida*.^31^ It has been shown to be applicable as a biosensor to detect gallic acid concentrations as low as 0.5–1 μM using a native host. However, this system has never found application outside *P. putida*. Therefore, the development of a broad-host range inducible system responding to gallic acid will be highly beneficial. It will be a valuable tool for engineering applications enabling to build the gallic acid-dependent synthetic regulatory circuitry, develop artificial biosynthetic pathways, for strain screening, and monitor this compound intracellularly or extracellularly.

In this study, we characterize an inducible gene expression system activated by the exogenous gallic acid. The analysis of gene cluster associated with gallic acid metabolism followed by genetic and functional examination enables identifying the 4-oxalomesaconic acid (4-OMA), a metabolic intermediate of gallic acid-metabolism, as a primary effector required for the activation of the inducible system. The inducible system is employed for the development of whole-cell biosensors using three different bacterial species. Developed biosensors are shown to be suitable for the detection of exogenous gallic acid. The specificity and sensitivity of *P. putida* and *E. coli*-based biosensors were evaluated, and their dynamics was parameterized. Finally, the whole-cell biosensor is applied for the detection of gallic acid in the extract from green tea leaves.

## Results and Discussion

### Gallic Acid-Inducible System from *P. putida* KT2440

**Figure 1:**
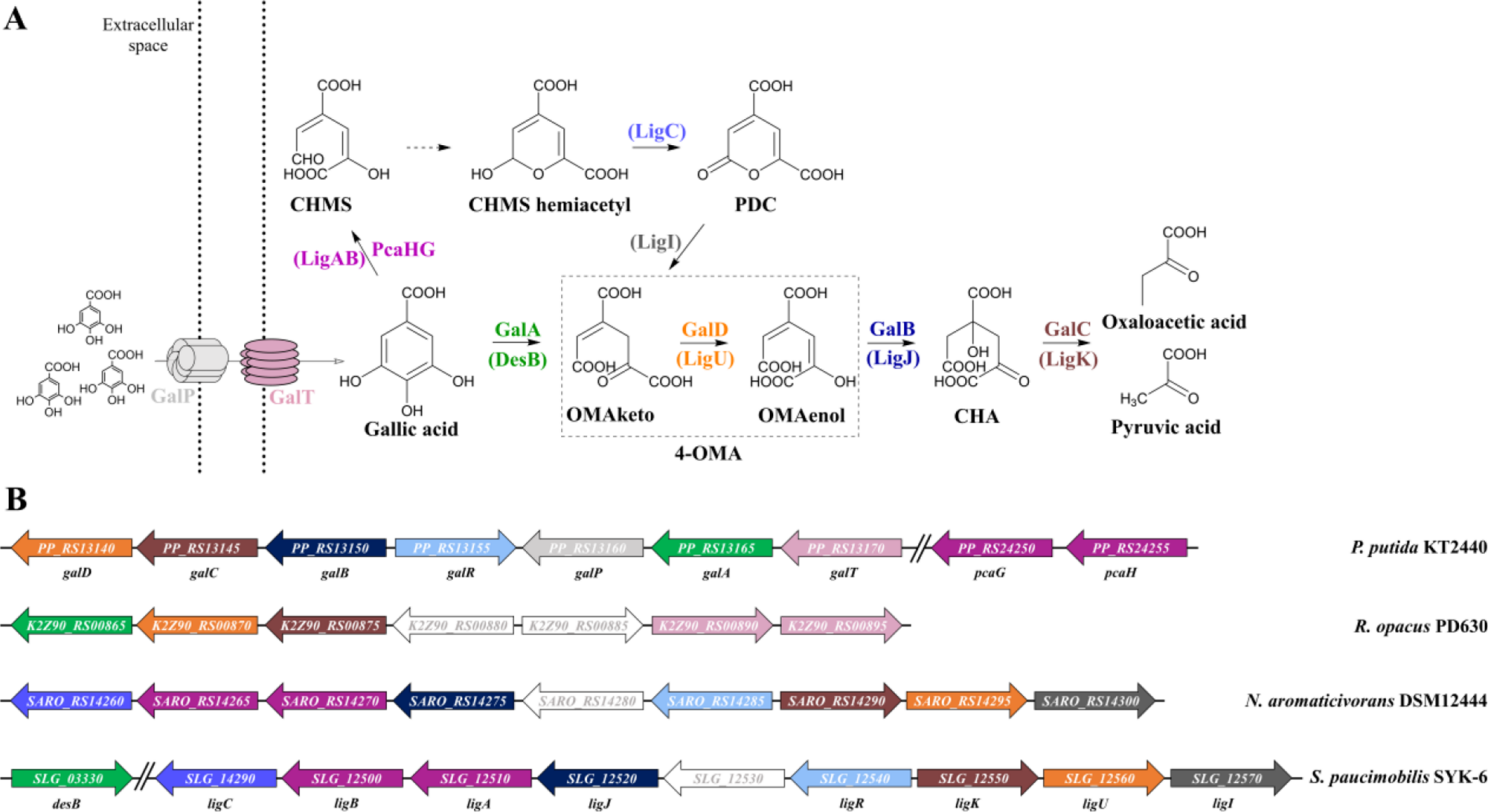
Gallic acid catabolic pathway. (A) Schematic representation of the gallic acid catabolic pathway in *P. putida*. Enzymes and transporters involved: 4-oxalomesaconic acid (4-OMA) tautomerase, GalD (orange); 4-carboxy-4-hydroxy-2-oxoadipic acid (CHA) aldolase, GalC (brown); oxalomesaconic acid enol (OMAenol) hydratase, GalB (dark blue); gallate dioxygenase, GalA (green); outer membrane porin, GalP (light gray); MFS transporter, GalT (pink). Enzymes catalyzing the same reactions in *S. paucimobilis* SYK-6 are shown in brackets. Catabolic intermediates: 2-pyrone-4-6-dicarboxylate, PDC; 4-carboxy-2-hydroxymuconate semialdehyde, CHMS (CHMS is converted to the hemiacetyl form nonenzymatically); isomers of 4-oxalomesaconic acid, OMAketo and OMAenol. (B) Genetic organization of the gene cluster encoding gallic acid catabolic pathway in members of actinomycetia (*R. opacus* PD630), α-(*S. paucimobilis* SYK-6, *N. aromaticivorans* DSM 12444), and γ-proteobacteria (*P. putida* KT2440). The color of genes matches the color code prescribed in (A); genes of unknown function are represented in white.

Catabolic pathways are often controlled by transcription factor-based inducible gene expression systems, which are composed of a transcriptional regulator (TR) and an inducible promoter that contains nucleotide sequences with TR and RNA polymerase (RNAP) binding motifs. Such inducible systems usually respond to the primary metabolic substrate, but sometimes they can be activated by the intermediate metabolic product.^30^ The gallic acid catabolic pathways have been characterized in *P. putida* KT2440^31^ (Figure 1A), *Sphingomonas paucimobilis* SYK-6,^32,33^*Novosphingobium aromaticivorans* DSM 12444,^34^ and *Rhodococcus opacus* PD630,^19^ and corresponding gene clusters have been identified (Figure 1B). A LysR family TR, known as GalR, has been shown to act as a transcriptional activator of *gal* operon in *P. putida* KT2440 when growth media have been supplemented with the gallic acid.^31^ The *gal* operon consists of *galA*, *galB*, *galC*, and *galD*, encoding enzymes responsible for the gallic acid degradation, along with *galT* and *galP* genes, aiding the gallic acid transport into the cell (Figure 1).

**Figure 2:**
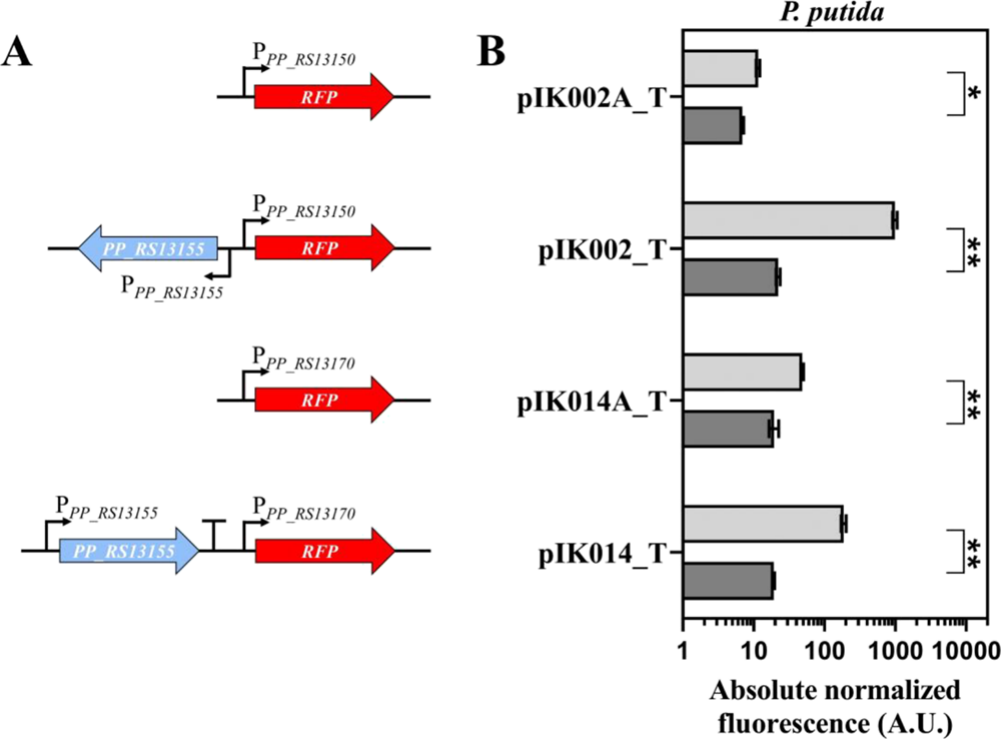
Evaluation of inducible gene expression systems responding to exogenous gallic acid in *P. putida* and analysis of genetic elements required for gene expression activation. (A) Genetic organization of inducible systems assembled in constructs pIK002A_T, pIK002_T, pIK014A_T, and pIK014_T. (B) Comparison of absolute normalized fluorescence of *P. putida* cells harboring constructs with different versions of inducible system. The absolute normalized fluorescence was determined using absorbance and fluorescence values measured 6 h after exogenous addition of gallic acid to the logarithmically growing cells in LB medium at a final concentration of 1.25 mM (light gray) or 0 mM (dark gray). Data represent mean values ± standard deviations (SD) of three biological replicates, *p < 0.01, **p < 0.001 (unpaired *t* test).

To evaluate the response of the inducible gene expression system from *P. putida* to gallic acid, plasmid constructs containing inducible systems composed of *galR* (*PP_RS13155*) and intergenic regions *PP_RS13150*/*PP_RS13155* (*galB*/*galR*) or *PP_RS13170*/*PP_RS13175* (*galT*/*glsB*) (Supplementary Figure S1A) were assembled in combination with the fluorescent reporter gene RFP (Figure 2A). The selected intergenic regions are adjacent to either *galB* or *galT* and contain promoters P_*PP_RS13150*_ or P_*PP_RS13170*_, respectively. *P. putida* KT2440 cells harboring constructs with putative inducible system variants were grown either in rich Luria–Bertani (LB) medium or minimal medium (MM), and single-time point fluorescence measurements of the logarithmically growing cells were performed 6 h after supplementation with the inducer to a final concentration of 1.25 mM (Figure 2B and Supplementary Figure S2). The highest level of RFP synthesis was observed for the pIK002_T containing *Pp*GalR/P_*PP_RS13150*_ inducible system mediating 45- or 248-fold induction in LB or MM medium, whereas the *P. putida* strain carrying a variant with promoter P_*PP_RS13150*_ (pIK002A_T) exhibited only a minor 1.7 or 6.3-fold increase of RFP synthesis, respectively, indicating that GalR TR is required for gene expression activation. Hereafter, *P. putida* KT2440 strain carrying pIK002_T with the *Pp*GalR/P_*PP_RS13150*_ inducible system was denoted as whole-cell biosensor BS1.

The putative inducible system *Pp*GalR/P_*PP_RS13170*_ (pIK014_T) and the promoter ‘only’ version P_*PP_RS13170*_ (pIK014A_T) both mediated the activation of gene expression, displaying approximately 9.7- and 2.5- or 72.3- and 45.6-fold induction in LB or MM medium, respectively. This shows that both promoters P_*PP_RS13150*_ and P_*PP_RS13170*_ are activated when gallic acid is exogenously added to the cell culture. The activation is further enhanced when cells are presented with an extra copy of *galR* gene on the plasmid confirming the GalR role as the transcriptional activator for both promoters and corresponding inducible systems *Pp*GalR/P_*PP_RS13150*_ and *Pp*GalR/P_*PP_RS13170*_. Since the former system exhibited a higher level of activation, it was used in the following evaluation and as a platform for developing whole-cell biosensors.

It should be noted that none of the GalR TR potential target binding sites have been characterized to date. To identify promoter P_*PP_RS13150*_ DNA regulatory elements, including TR binding site(s) and RNA polymerase (RNAP)-35 and -10 binding sites along with transcription start site (TSS), bioinformatics analysis of intergenic region *PP_RS13150*/*PP_RS13155* was performed. First, DNA sequences of two intergenic regions *PP_RS13150*/*PP_RS13155* and *PP_RS13170*/*PP_RS13175* from *P. putida* KT2440 were compared using Multiple Sequence Alignment tool Clustal Omega (Supplementary Figure S1A). Second, the RNAP-35 and -10 binding sites and TSS were predicted using SAPPHIRE.^35^ Finally, by multiple species sequence alignment, the conserved functional elements were distinguished from nucleotide sequences that were less essential and prone to evolve rapidly. To this end, the nucleotide sequences of intergenic region *PP_RS13150*/*PP_RS13155* from 40 *Pseudomonas* species with GalR protein sequence identities of at least 60% were aligned to obtain a sequence similarity logo (Supplementary Figure S1B), revealing promoter P_*PP_RS13150*_ regulatory elements.

LysR-type TR typically interacts with two distinct binding sites, a regulatory binding site (RBS) often found upstream RNAP binding region and an activation binding site (ABS), located near the −35 binding site, in the presence of an effector subsequently interacting with RNAP and inducing target gene expression.^36^ Examination of intergenic region *PP_RS13150*/*PP_RS13155* revealed a conserved nearly 40-nucleotide sequence region between positions −62 and −97 upstream to the *galB* start, which contains two motifs that resemble RBS and ABS sites.

The predicted RBS contains the typical LysR T-N_11_-A core motif^37^ and to large degree resembles the extended version of this motif, CTATA-N_9_-TATAG.^38^ While the ABS is less conserved, it contains 7-nucleotide sequence AACGCAT that is identical to the right part of the dyad RBS motif. Typically to LysR-type regulatory elements, the GalR ABS is near the RNAP -35 binding site.

We conclude that the effector-bound GalR TR potentially binds RBS and ABS regulatory elements mediating RNAP interaction with promoter and transcription activation. The predicted architecture of regulatory elements enabling the molecular mechanisms of TR GalR-promoter P_*PP_RS13150*_ interaction and inducible system *Pp*GalR/P_*PP_RS13150*_ activation are summarized in Supplementary Figures S1 and 3. This information will be useful for synthetic biology and genetic parts design to advance the research on the gallic acid metabolism and production.

**Figure 3:**
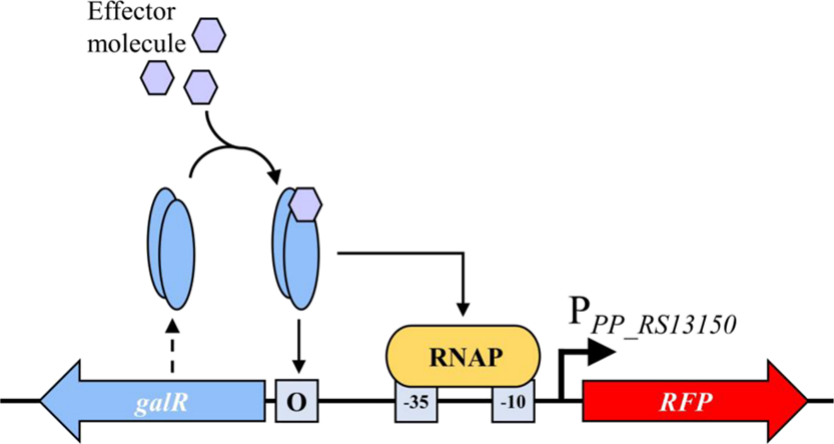
Schematic representation of inducible system *Pp*GalR/P_*PP_RS13150*_ coupled to fluorescent reporter, RFP. LysR-type TR GalR enables the active RNA polymerase-promoter complex formation and initiation of the reporter gene expression, when the effector molecule is present and it is bound to the TR.

### Development of *E. coli* and *C. necator*-Based Whole-Cell Biosensors

**Figure 4:**
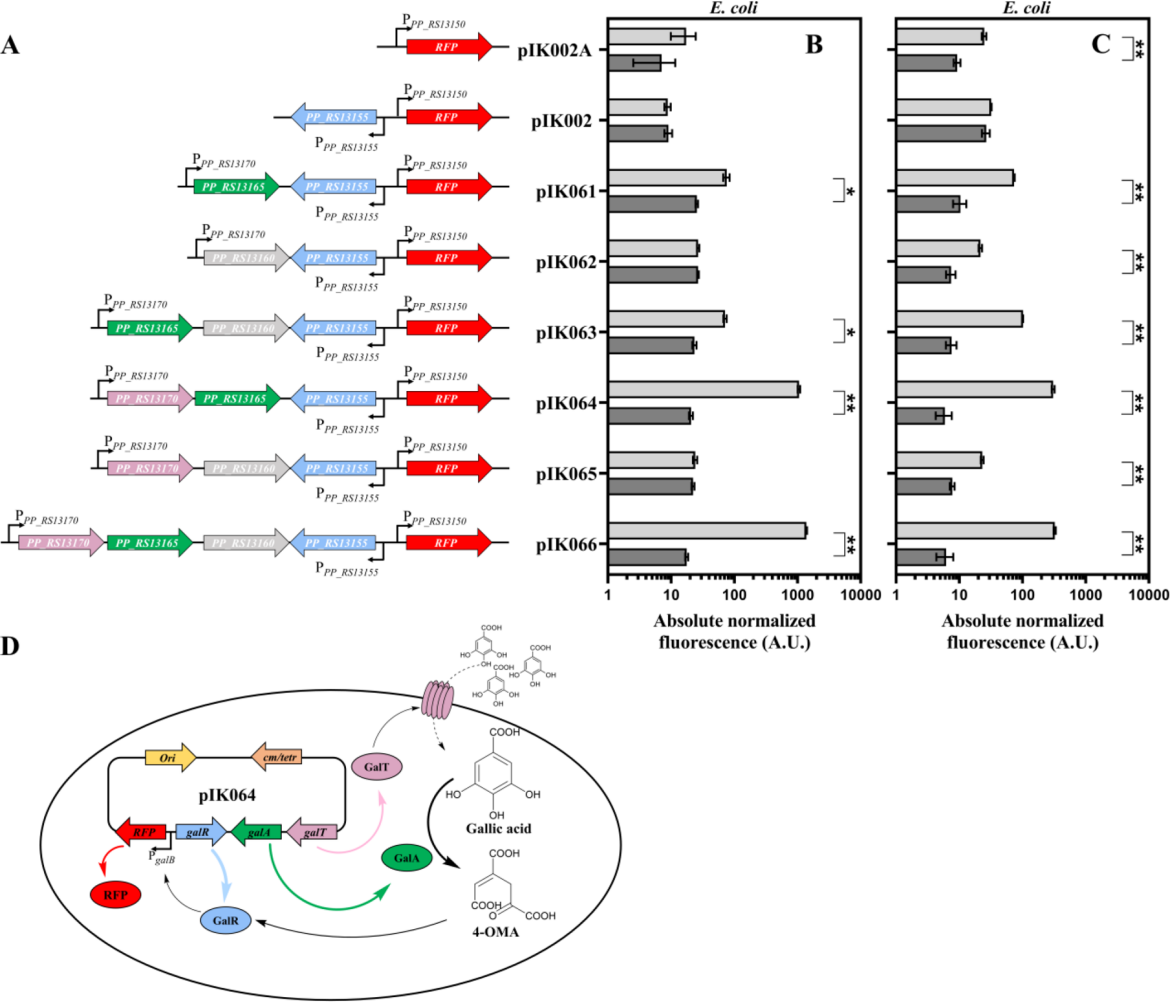
Development of the gallic acid biosensor based on non-native host *E. coli* Top10. (A) Genetic organization of inducible system’s variants supplemented with different sets of additional genes including *galP*, *galA* and *galT*. Results represent absolute normalized fluorescence measured in LB (B), and MM medium (C) 6 h after exogenous addition of gallic acid to the final concentration of 5 mM (light gray) or 0 mM (dark gray), Data represent mean values ± SD of three biological replicates, *p < 0.01, **p < 0.001 (unpaired *t* test). (D) Principal scheme and activation mechanism of *E. coli* -based biosensor BS2.

In this study, aiming to develop a broad-host range inducible system that can be used for gallic acid detection in nonhost microorganisms, the *Pp*GalR/P_*PP_RS13150*_ inducible system was subjected to testing in γ-proteobacterium *E. coli* and β-proteobacterium *C. necator*. However, neither *E. coli* nor *C. necator* carrying *Pp*GalR/P_*PP_RS13150*_ inducible system (pIK002) exhibited increase of RFP synthesis in the presence of gallic acid (Figure 4A–C and Supplementary Figure S3A–C).

The genes of TRs including LysR family regulators are commonly located next to and oriented in the opposite direction to the genes or operons, which they control.^36,39^ Often, the primary effector that interacts with TR and activates the gene expression is metabolized by the operon-encoded enzymes. As the *galB* gene encoding 4-OMA hydratase is transcribed in the divergent orientation and adjacent to *galR*, we hypothesized that 4-OMA might be the effector that binds the GalR TR, inducing the gene expression. Moreover, we reasoned that the gallate dioxygenase GalA, characterized previously in *P. putida*,^40^ as well as outer membrane porin GalP and major facilitator superfamily (MFS) transporter GalT is required to convert gallic acid into 4-OMA and to enable the efficient gallic acid transport into the cell. To test our hypothesis, constructs containing *Pp*GalR/P_*PP_RS13150*_ inducible system coupled with different combinations of genes *galA*, *galP,* and *galT* were assembled (Figure 4A). These constructs were then investigated in *E. coli* and *C. necator* cells for the inducible system’s response to the exogenous supplementation of gallic acid.

Remarkably, *E. coli* strains carrying inducible systems with a copy of *galA* gene (pIK061, pIK063, pIK064, and pIK066) exhibited a significant activation of reporter gene expression when the cell cultures were exogenously supplemented with the gallic acid. Constructs pIK064 and pIK066, both containing common genes *galT*, *galA*, and *galR* and promoter P_*PP_RS13150*_, mediated the highest induction (51- and 78-fold, respectively), resulting in an increase of absolute normalized fluorescence from approximately 20 to more than 1000 AU 6 h after addition of gallic acid at a final concentration of 5 mM (Figure 4B). A similar level of activation was observed when the cells were grown in MM supplemented with the gallic acid (Figure 4C). Subsequently, *E. coli* strain carrying plasmid pIK064 with *Pp*GalTGalAGalR/P_*PP_RS13150*_ module was denoted as whole-cell biosensor BS2. Curiously, the inclusion of *galT* gene (pIK064) manifested in the enhanced RFP synthesis in *E. coli*. A protein similarity search revealed that coincidently *E. coli* possesses no homologs of GalT. This suggests that the *P. putida* GalT transporter is functional and improves the gallic acid uptake in *E. coli*.

In addition, a 2.25-fold activation of reporter gene expression in *C. necator* resulting in an increase of absolute normalized fluorescence from approximately 60 to 135 AU in LB medium when the copy of *galA* gene was included (pIK061) (Supplementary Figure S3B). Notably, no stable *C. necator* was possible to achieve with plasmids pIK062, pIK063, pIK064, pIK065, and pIK066. In addition, plasmid loss was observed when pIK061-transformed cells were grown in MM (Supplementary Figure S3). The attempts to achieve stable transformation of *P. putida* with constructs containing an additional copy of different combinations of *galP*, *galA*, and *galT* genes was only partially successful (Supplementary Figure S4). Overall, the data obtained using constructs pIK061, pIK062, pIK063, and pIK065 showed similar levels of reporter gene expression activation in the presence of gallic acid as for *P. putida* carrying pIK002.

Considering that the *galA* encodes gallate dioxygenase catalyzing conversion of gallic acid into 4-OMA and that the *E. coli* is not known to possess the OMAenol hydratase (*galB*) or any other enzyme involved in the degradation of this compound, our results suggest that the 4-OMA is a primary effector likely interacting with GalR TR and mediating the induction of gene expression (Figure 4D). Further research will be required to elucidate whether keto, enol or both forms of 4-OMA act as an effector of *Pp*GalR/P_*PP_RS13150*_ inducible system. Interestingly, the protein similarity search revealed that the 4-oxalomesaconate tautomerase homologs exist in *E. coli* (*b0769*) and *C. necator* (*H16_RS33685*), exhibiting 48 and 45% sequence identity to *P. putida* GalD, respectively, which can potentially contribute to interchange between the OMAketo and OMAenol. Notably, when the action of GalD is omitted from the reaction’s order, only the enol form (OMAenol) and not the keto (OMAketo) is used as a substrate by GalB (OMAenol hydratase) in *P. putida* KT2440.^41^ However, based on the protein similarity search, neither *E. coli* nor *C. necator* possess GalB homologs, indicating that 4-OMA cannot be metabolized in these bacterial species.

### Biosensor Specificity

**Figure 5:**
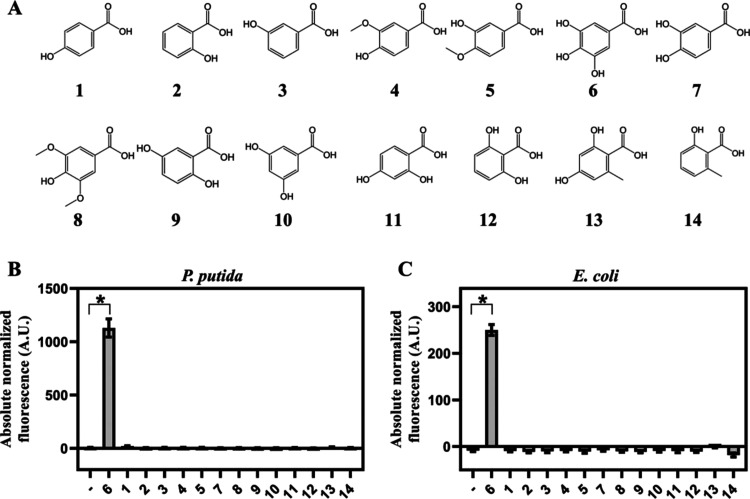
Specificity of gallic acid-inducible biosensors. (A) Compounds that were tested for cross-reactivity with gallic acid-inducible systems: *p*-hydroxybenzoic acid (1), salicylic acid (2), *m*-hydroxybenzoic acid (3), vanillic acid (4), isovanillic acid (5), gallic acid (6), protocatechuic acid (7), syringic acid (8), gentisic acid (9), α-resorcylic acid (10), β-resorcylic acid (11), γ-resorcylic acid (12), orsellinic acid (13), and 6-methylsalicylic acid (14). Absolute normalized fluorescence of BS1 (B) and BS2(C) cultivated in LB medium 6 h after supplementation with different hydroxybenzoic acids to the final concentration of 1.25 mM. Data represent mean values ± SD of three biological replicates, *p < 0.001 (unpaired *t* test).

Biosensors BS1 (*P. putida/Pp*GalR/P_*PP_RS13150*_) and BS2 (*E.coli*/*Pp*GalTGalAGalR/P_*PP_RS13150*_) were investigated for cross-activation with other major hydroxybenzoic acids (Figure 5A). The absorbance and fluorescence outputs of BS1 and BS2, cultivated in LB medium, were monitored overtime after the addition of different hydroxybenzoic acids to a final concentration of 1.25 mM (Supplementary Figure S5). Absolute normalized fluorescence levels were estimated 6 h after addition of compounds to BS1 (Figure 5B) and BS2 (Figure 5C). For both biosensors tested, neither of analyzed hydroxybenzoic acids except gallic acid mediated a significant induction of reporter gene expression. Therefore, we can conclude that both biosensors, BS1 and BS2, do not display any cross-reactivity with compounds from the same group and are specific to gallic acid.

### Parameterization of Biosensors

**Figure 6:**
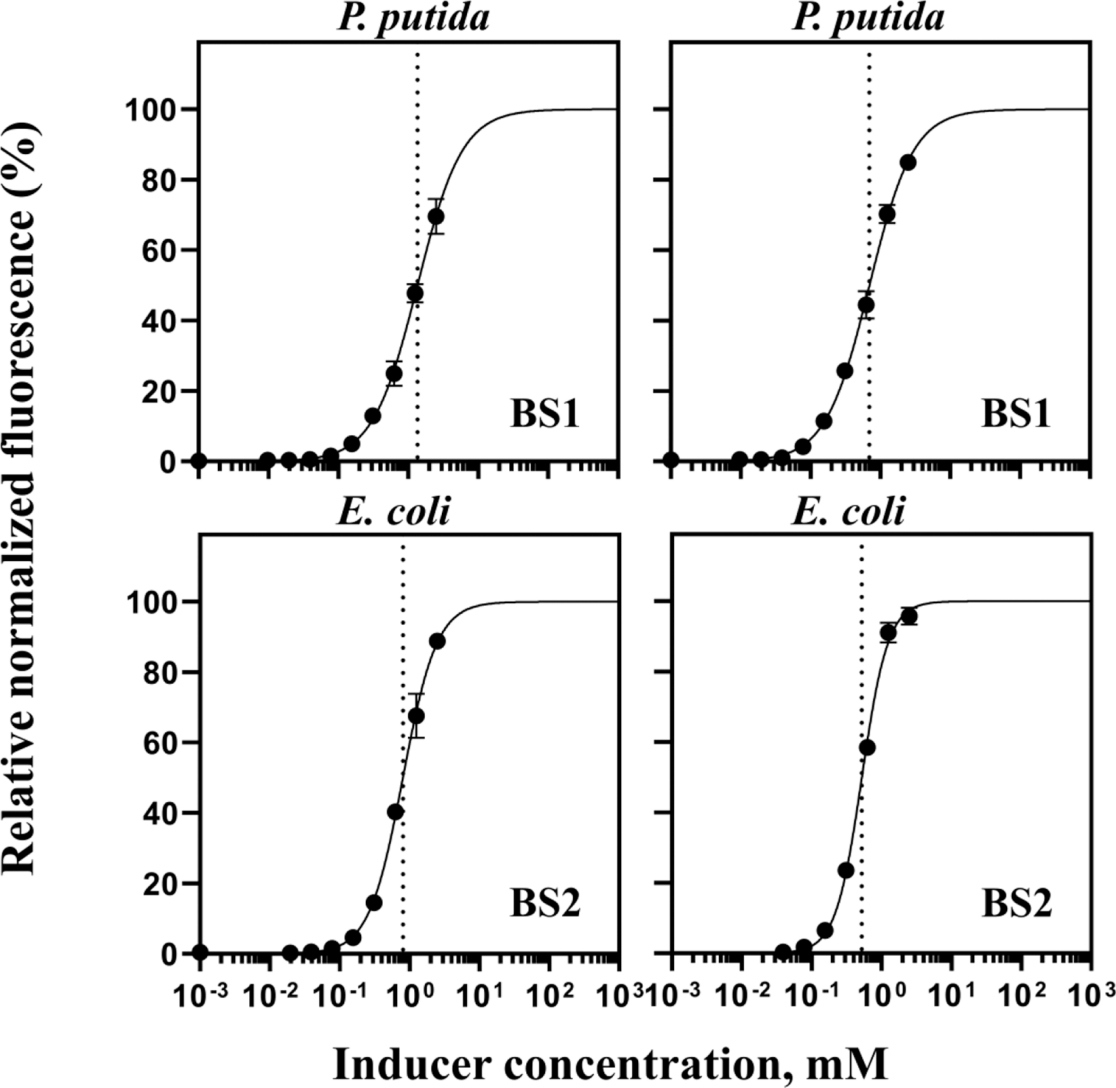
Dose–response curves of gallic acid-inducible biosensors. Relative normalized fluorescence of BS1 and BS2 6 h (left) and 12 h (right) after addition of different concentrations of gallic acid, ranging from 0 to 2.5 mM. The dose–response curves were fitted using the Hill function as described in the Methods section. *K*_m_ is indicated by a dotted line. Data represent mean values ± SD of three biological replicates.

*P. putida* and *E. coli*-based biosensors BS1 and BS2 were further characterized by monitoring their response 6 and 12 h after supplementation with different concentrations of gallic acid ranging from 0 to 2.5 mM. Figure 6 shows the relationship between the concentration of extracellularly added gallic acid and fluorescence output. The curves of both tested biosensors indicate that the gene expression can be tuned in the range of approximately 0.312 to 1.25 mM. The lowest concentration of gallic acid activates the biosensor significantly, representing a limit of detection of 9.7 and 78 μM for BS1 and BS2, respectively. Unfortunately, gallic acid is prone to oxidation and polymerization^42^ forming polyphenol of dark color^31,19^ that interferes with absorbance measurements and limits the use of this compound to a relatively low concentration. Notably, the growth of both bacterial strains was similar for all concentrations of gallic acid tested (Supplementary Figure S6).

Nevertheless, biosensor BS1 exhibited dynamic ranges of approximately 112- and 2138-fold, whereas BS2 demonstrated approximately 75- and 162-fold induction 6 and 12 h, respectively, after the addition of gallic acid to logarithmically growing bacterial cells in LB medium (Table 1). The higher dynamic ranges with the *P. putida*-based BS1 biosensor likely indicate a more efficient conversion of gallic acid forming 4-OMA, whereas the introduction of exogenous and incomplete gallic acid-metabolic pathway into nonhost microorganisms such as *E. coli* (BS2) potentially affects cellular homeostasis and pathway regulation, prompting a limited induction of gene expression. Notably, both biosensors BS1 and BS2 exhibit similar *K*_m_ values and overall dynamics.

**Table 1:** Parameters of *E. coli* and *P. putida*-Based Whole-Cell Biosensors, Measured in LB Medium^a^

whole-cell biosensor	time, h	*b_max_*	*b_min_*	dynamic range, -fold	*K_m_*, mM	Hill’s coefficient
BS1	6 h	989.5	9.873	112 ± 40	1.459 ± 0.44	1.381 ± 0.14
BS1	12 h	1681	1.128	2138 ± 1735	0.706 ± 0.09	1.394 ± 0.07
BS2	6 h	327.5	5.165	75 ± 32	0.868 ± 0.19	1.817 ± 0.32
BS2	12 h	498.4	3.343	162 ± 40	0.525 ± 0.01	2.321 ± 0.13

a Data are mean ± SD, *n* = 3.

Additionally, the response of biosensors BS1 and BS2 to concentrations of gallic acid ranging from 0 to 1.25 mM were evaluated in MM medium (Supplementary Figure S7). The dose–response curves of BS1 indicate that the gene expression can be tuned in the range from 0.039 to 1.25 mM, whereas curves of BS2 specify the tunable range from 0.078 to 1.25 mM (Supplementary Figure S8). The lowest concentrations of gallic acid for significant activation of BS1 and BS2 were 2.44 and 39 μM, respectively. This indicates the capacity of both biosensors to measure gallic acid at lower concentrations in MM medium, suggesting a wider applicability scope. In addition, induction rates of BS1 were 246- and 263-fold, while BS2 displayed 20- and 124-fold induction 6 and 12 h after addition of various concentrations of gallic acid, respectively (Supplementary Table S1).

Finally, it should be noted that the previously reported gallic acid concentrations obtained in the metabolically engineered microorganisms ranges from 0.35 to 117 mM^12,16−19^ can be detected using the biosensors developed in this study.

### Application of the Biosensor for Determination of Gallic Acid in Green Tea Extract

Application of biosensors can help to improve the speed and limit of detection, directly determining the concentration of a bioactive molecule or indirectly monitoring interactions with biological matter, regardless of the molecule’s chemical structure.^43^ Thus far the detection of gallic acid in green tea samples was reported using diverse electrode-based methods. However, they are usually sophisticated, nonspecific,^44^ displaying limited stability,^45^ and exhibiting low-sensitivity as well as lacking the ability for real-time and high-throughput measurements.

The green tea is known to be rich in catechins that includes gallic acid residue in their structure.^46^ The epigallocatechin-3-gallate (EGCG) is most abundant catechin that was shown to degrade into gallic acid and other catechins in aqueous solutions or in green tea infusions upon heat treatment. Another catechin, the epicatechin gallate (ECG), found in green tea is present in a lesser quantity and it is more stable. The stability of EGCG and the emergence of degradation products were shown to be concentration-dependent, demonstrating higher stability in higher concentrations. Notably, it was reported that the amount of gallic acid increased with decreasing EGCG concentration.^47^ In this study, the gallic acid release through the degradation of EGCG and ECG was assessed by using HPLC-UV. Notably, almost all EGCG was degraded and the free gallic acid was accumulated after 24 h of incubation in MM at 30 °C (Supplementary Figure S9), whereas the amount of ECG reduced only by 50%, and no gallic acid was formed.

Consequently, we reasoned that the nonenzymatic degradation of EGCG followed by the conversion of gallic acid into the 4-OMA by gallate dioxygenase would enable utilizing the developed biosensor to determine the free gallic acid in green tea extract. To ensure that the BS1 responds to exogenous gallic acid, and is not activated by any of the catechins that contain residue in their structure, the absolute normalized fluorescence was determined in BS1 cultures in MM supplemented with 5 mM EGCG and ECG (Supplementary Figure S10). Only the EGCG sample exhibited delayed induction indicating that the biosensor responds to the gallic acid released through EGCG degradation. These results confirmed that neither EGCG nor ECG activates the biosensor.

To determine the amount of gallic acid in green tea, the extraction was performed by the infusion method, and the lyophilized powder of green tea extract was prepared as described in the Methods section. The extraction yield of soluble residue was 11.21% of green tea dry weight (DW). It was previously reported that the amount of gallic acid and EGCG in green Chinese tea range from 0.068 to 0.168 and 4.943 to 10.196 (% w/w), respectively,^46^ whereas Tan et al. showed that 8.65 mg of gallic acid/g DW can be obtained from green tea extract using the infusion method.^48^

**Figure 7:**
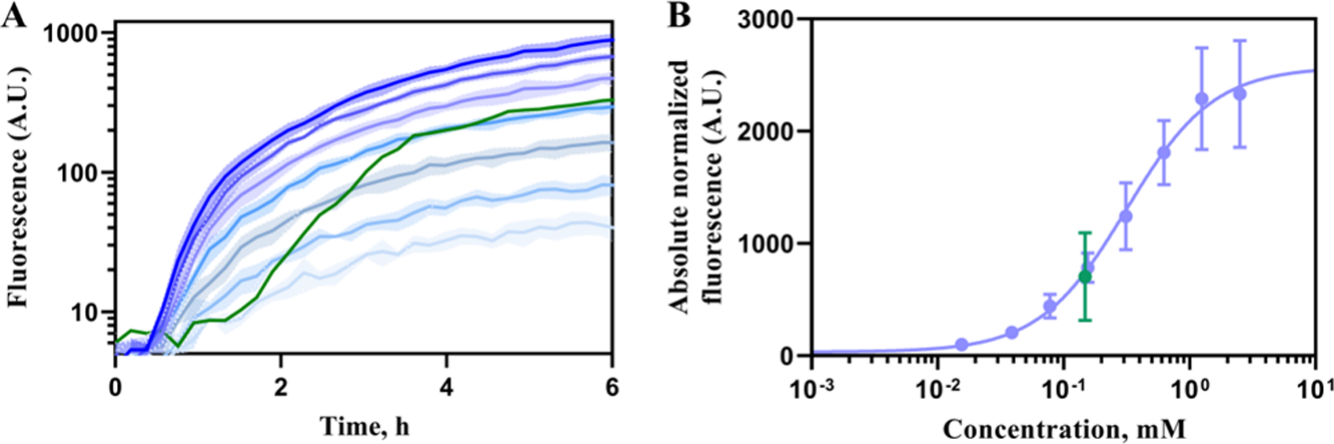
Application of BS1 for determination of gallic acid in green tea extract. (A) Absolute normalized fluorescence of BS1 in MM supplemented with green tea extract at the final 160-fold dilution (green) and 0.0195, 0.039, 0.078, 0.156, 0.312, 1.25, and 2.5 mM gallic acid (blue), the darker the color shade the higher concentration was added. (B) Dose–response curve fitted using the Hill function was used for gallic acid estimation in green tea extract. Values of gallic acid standards are in blue, while the green tea extract is in green. Data represent mean values ± SD of three biological replicates.

The RFP fluorescence output of BS1 culture supplemented with the 160-fold diluted green tea extract was compared to the induction with different concentrations of gallic acid in MM (Figure 7A). A delay in the biosensor response was observed when the green tea extract was used indicating that the generation of gallic acid from EGCG was required for activation. By applying a nonlinear least-squares fitting to the fluorescence outputs obtained 6 h after the addition of different concentrations of gallic acid to BS1 culture in MM, the equation of Hill function was derived with the *K*_m_ value of 0.4372 mM and Hill coefficient of 1.4. The absolute normalized fluorescence value of green tea extract was then fitted to this equation and the concentration of gallic acid was determined as 0.148 mM (Figure 7B), representing 23.71 mM undiluted sample and 24.2 mg/g DW green tea extract or 2.71 mg/g DW green tea. This yield of gallic acid is lower but comparable to previously reported 8.65 mg of gallic acid/g DW obtained from green tea extract using the infusion method.^48^ To compare the sensitivity of analytical methods, the concentration of free gallic acid was measured with biosensor BS1 or by HPLC-UV using samples of green tea extract that was diluted from 20 to 2560-fold. Only samples of up to 100-fold diluted extract contained a sufficiently high concentration of gallic acid, which can be accurately quantified by HPLC, despite that with pure gallic acid sample a resolution of up to 0.0625 mM was achieved (Supplementary Figure S11), whereas biosensor exhibited statistically significant activation of reporter expression with up to 1280-fold diluted extract sample. This result shows that the developed biosensor can be used not only for the detection of gallic acid, but it offers a significant improvement of detection limit compared to the HPLC method in complex mixtures such as green tea extract.

## Methods

### Chemicals, Bacterial Strains, and Media

Chemicals used as inducers for assaying whole-cell biosensors are listed in Supplementary Table S2. All strains used in this study are presented in Supplementary Table S3. *E. coli* was used for cloning and plasmid propagation. Fluorescence assays were performed using *E. coli*, *P. putida*, or *C. necator* as hosts, and genomic DNA of *P. putida* was used as a template to PCR amplify DNA fragments containing gallic acid-inducible system’s genetic elements. Cells were cultivated in Luria–Bertani (LB) medium, or MM medium (*E. coli* Top10: M9 medium supplemented with 2 mM MgSO_4_, 0.1 mM CaCl_2_, 1 μg/mL thiamine hydrochloride, 0.4 mM L-leucine, and 0.2% (w/v) glycerol,^49^*P. putida* KT2440: M9 medium supplemented with 2 mM MgSO_4_, 0.1 mM CaCl_2_, 1 μg/mL thiamine hydrochloride, 0.4 mM L-leucine, and 0.4% (w/v) glucose,^50^*C. necator*: SL7 solution supplemented with 25 mM Na_2_HPO_4_ × 12 H_2_O, 11 mM KH_2_PO_4_, 18.7 mM NH_4_Cl, 0.8 mM MgSO_4_ × 7 H_2_O, 0.14 mM CaCl_2_ × 2 H_2_O, 4.6 μM Fe(*III*)NH_4_-Citrate, and 0.4% (w/v) sodium gluconate),^51^ whereas for assaying green tea induction levels in *P. putida*, MM medium was used. Antibiotics were added to the medium at the following concentrations: 25 μg/mL or 50 μg/mL chloramphenicol for *E. coli* and *C. necator*, respectively, and 10 μg/mL tetracycline for *P. putida*. The solid medium was prepared by supplementation with 15 g/L agar.

### Cloning and Transformation

Plasmid DNA was purified by using the GeneJET Plasmid Miniprep Kit (Thermo Fisher Scientific). Microbial genomic DNA was extracted by employing the GenElute Bacterial Genomic DNA Extraction Kit (Sigma-Aldrich). To derive the gel-purified linearized DNA, a Zymoclean Gel DNA Recovery Kit (Zymo) was used. Phusion High-Fidelity DNA polymerase, DreamTaq DNA polymerase, restriction enzymes, and T4 DNA Ligase were purchased from Thermo Fisher Scientific. NEBuilder HiFi DNA assembly kit was purchased from New England Biolabs. All reactions were set up according to the manufacturer’s protocol. Chemically competent *E. coli* were prepared and transformed using a heat-shock method as described by.^50^ Electrocompetent *C. necator* and *P. putida* were prepared and transformed using the electroporation method as described in ref^52^. For transferring the plasmids into *P. putida*, the antibiotic resistance gene was changed from chloramphenicol to tetracycline by using oligonucleotide primers IK003 and IK004 to amplify the tetracycline resistance gene from pME6000 and cloned by AscI and PmeI restriction sites into the required plasmids.

### Plasmid Construction

Plasmids were constructed using restriction enzyme and ligation-based method^50^ or NEBuilder HiFi DNA assembly master mix according to the manufacturer’s protocol (New England Biolabs) by cloning PCR amplified DNA fragments into the pBRC1 vector,^53^ which was built as described for pEH006 in.^54^

To construct plasmid pIK002, oligonucleotide primers EV001-PP_2515 and EV003-PP_2515B were used to PCR-amplify putative gallic acid-inducible system (gene *galR* and intergenic region *galR-galB* (*PP_RS13155*-*PP_RS13150*) from *P. putida* KT2440 genomic DNA). For plasmid pIK002A, primer pair EV001A/EV003-PP_2515B was used to amplify intergenic region *galR-galB*. All amplified DNA fragments were prepared by digestion with AatII and NdeI restriction endonucleases (Thermo Fisher Scientific) and cloned by ligation into pBRC1 vector through AatII and NdeI restriction sites.

Plasmids pIK014A, pIK014, pIK061, pIK062, pIK063, pIK064, pIK065, and pIK066 were constructed by employing the NEBuilder HiFi DNA assembly method. pBRC1 was linearized with AatII and NdeI restriction endonucleases and used as a cloning vector and *P. putida* KT2440 genomic DNA was used as a template for PCR amplification. To construct plasmid pIK014, oligonucleotide primer pairs EV001B/EV003C and EV008-PP/EV009-PP were used to amplify gene *galR* and intergenic region *galR-galT* (*PP_RS13155*-*PP_RS13170*). For plasmid pIK014A, primer pair EV008B-EV009-PP was used to amplify intergenic region *galR-galT*. To assemble plasmid pIK061, primer pairs EV001E/EV003-PP_2515B, EV008B/IK025, and IK023/IK024 were used to amplify genes *galA* (*PP_RS13165*) and *galR,* and intergenic region *galR-galB*. For pIK062, primer pairs EV008B/IK025 and EV003-PP_2515B/IK026 were used to amplify genes *galP* (*PP_RS13160*) and *galR,* and intergenic region *galR-galB*. To construct plasmid pIK063, primer pairs EV008B/IK025 and EV003-PP_2515B/IK023 were used to amplify gene cluster *galAPR,* and intergenic region *galR-galB*). For plasmid pIK064, primer pairs EV008B/IK024 and EV003-PP_2515B/EV001E were used to amplify genes *galT* (*PP_RS13170*), *galA* and *galR,* and intergenic region *galR-galB*. To assemble pIK065, primer pairs EV008B/IK027 and EV003-PP_2515B/IK028 were used to amplify *galT*, *galP*, *galR,* and intergenic region *galR-galB*. For pIK066, primers EV008B and EV003-PP_2515B were used to amplify gene cluster *galTAPR,* and intergenic region *galR-galB*.

The validation of plasmids was performed by colony PCR and restriction-based analysis. Oligonucleotide primers were synthesized by Metabion International AG, and they are listed in Supplementary Table S4.

### Extraction of Gallic Acid from Green Tea

Green tea (*Camellia sinensis*) leaves were purchased from a local market (“Chinese Green Tea”), and the tea leaves were ground until they turned into the powder suspension to enhance the extraction process. Then, 10 g of the powder was infused with 100 mL of distilled water at 100 °C for 5 min and then kept for 4 h at room temperature. The entire content was transferred to 50 mL falcon tubes and centrifuged at 11,000 rpm for 5 min. Resulting supernatant was then filtered using filter paper, frozen at −80 °C overnight and subsequently freeze-dried under vacuum conditions, at −78 °C for 48 h to remove H_2_O using the Scientific Freeze Dryer (SP Industries). The freeze-dried extract was stored in amber glass bottles at −80 °C before HPLC and fluorescence analysis was performed. The yield of extraction was estimated using the following formula 1:
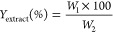
1where *W*_1_ is the weight of extract residue obtained after solvent removal and *W*_2_ is the weight of grounded tea leaves.

The yield of gallic acid extracted from green tea leaves was estimated by dissolving 1 mg/mL of lyophilized extract in water, and subjecting to HPLC analysis, where retention times were matched with standards from calibration curves. The quantity of detected gallic acid was identified using regression equation and expressed as g/100 g DW.

### HPLC Analysis

HPLC analysis of gallic acid and EGCG was performed using an Ultimate 3000 HPLC system equipped with a photodiode array (UV–VIS) detector (Thermo Fisher Scientific). Chromatographic separation was achieved with a Phenomenex Luna 5 μm C18 100 Å (150 × 4.60 mm) column equipped with a Phenomenex Security Guard Cartridge (part number KJ0-4282), thermostated at 25 °C. The mobile phase A was aqueous 0.1% formic acid (v/v); and mobile phase B was HPLC-grade acetonitrile. The elution gradients used were as follows: from 0 until 15 min from 10 to 50% B, from 15–17.5 min raised at 70% B; 17.5–20 min decreased to 10% B then kept constant for 2 min. A constant flow rate of 1 mL/min was kept throughout the analysis with the detection wavelength set at 260 nm. The samples were diluted five times with water and then filtered using a 0.22 μm syringe filter. Ten microliters of sample were injected, and the elute was detected at a wavelength of 280 nm. All chromatograms were recorded and analyzed using Chromeleon 7 software (Thermo Fisher Scientific, USA). Gallic acid and EGCG solutions with concentrations ranging from 0.0625 to 2 mM and from 0.031 to 1 mM, respectively, were used as standards to generate calibration curves for estimation of compound concentrations in extract samples. The dilutions of green tea extract ranging from 20 to 2560-fold were used for the determination of concentration and limit of detection for gallic acid.

### Absorbance and Fluorescence Assessments

For quantification of absolute normalized fluorescence, plasmid-transformed bacterial cells were grown overnight in 2 mL of LB medium or MM medium containing appropriate antibiotic with orbital shaking at 200 rpm and 30 °C. *P. putida*, *E. coli*, and *C. necator* cell cultures were then diluted, 60, 50, and 50 times, respectively, into a fresh LB medium or 20 times into MM medium with respective antibiotic and they were grown with 200 rpm orbital shaking at 30 °C in 50 mL conical tubes. The 142.5 μL of exponentially growing cells with an absorbance *A*_600_ of 0.05–0.2 were transferred to a 96-well microtiter plate (flat and clear bottom, black, Fisher Scientific) and supplemented with 7.5 μL of inducer to achieve a final concentration as indicated. RFP fluorescence was determined using an Infinite M200 PRO (Tecan, Austria) microplate reader. The RFP fluorescence was measured using 585 nm as excitation wavelength and 620 nm as emission wavelength, with 9 nm and 20 nm bandwidths, respectively. The gain factor was set to 120%. Simultaneously, the absorbance was measured using a wavelength of 600 nm with a 9 nm bandwidth. The obtained values, when gallic acid, ECG and EGCG were used as source of inducer, were normalized by calculating absolute normalized fluorescence (ANF) as described previously^55^2:
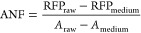
2where RFP_raw_ and *A*_raw_ are absolute fluorescence and absorbance values of culture; RFP_medium_ and *A*_medium_ – absolute fluorescence and absorbance values of the medium.

For the analysis of gallic acid content in green tea, due to the dark color of extracts, the fluorescence normalization was performed using the fluorescence and absorbance of the medium supplemented with the same amount of extract.

### Parametrization of the Biosensor

Biosensors were parametrized by applying a nonlinear least-squares fitting to the Hill function. The values of ANF were calculated and plotted as a function of effector concentration using the software GraphPad Prism 9 and the following eq 3:
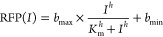
3where RFP(*I*) is the absolute normalized fluorescence value at given effector concentration *I*; *b*_max_ and *b*_min_ are the maximum and minimum levels of reporter output in absolute normalized fluorescence units, respectively; *h* is the Hill coefficient; *K*_m_ is the inducer concentration, corresponding to the half-maximal reporter’s output.

The dynamic range indicating the fold of induction was calculated by dividing either ANF of induced sample by ANF of uninduced sample or *b*_max_ by *b*_min_, when the latter parameters were estimated for the dose–response analysis.

### Determination of Gallic Acid in Green Tea

The concentration of gallic acid in green tea extract was determined as follows; 7.5 μL of green tea extract at different dilutions or gallic acid standards at different concentrations were added to the 142.5 μL of exponentially growing BS1 in MM medium at the absorbance *A*_600_ of 0.05–0.2. The dilutions of green tea extract ranged from 20 to 2560-fold. The concentrations of gallic acid standards were 0, 0.0195, 0.039, 0.078, 0.156, 0.312, 1.25, and 2.5 mM. 150 μL BS1 cultures supplemented with either green tea extract or gallic acid were transferred into the 96-well microtiter plate followed by the absorbance and RFP fluorescence measurements using an Infinite M200 PRO (Tecan, Austria) microplate reader as described above. ANF data obtained with the gallic acid standards were used for the nonlinear least-squares fitting of dose–response curve by applying the Hill function as described above. The obtained equation and ANF values determined for green tea extract were then used to estimate the concentration of gallic acid.

### Statistical Analysis

All data presented in this study are mean ± SD, *n* = 3. The results were analyzed using GraphPad Prism 9.0, using an unpaired two-tailed *t* test to compare the means, the *p*-values of 0.01 or 0.001 were considered significant.

### Determination of the Consensus Sequence and Prediction of Regulatory Elements

The nucleotide sequences of *PP_RS13150*/*PP_RS13155* intergenic regions were aligned by using Multiple Sequence Alignment tool (Clustal Omega).^56^ A sequence similarity motif was generated using WebLogo.^57^ Putative RNAP-10 and -35 binding sites and TSS were predicted by using SAPPHIRE.^35^

## Supporting Information Available

Chemicals, bacterial strains, oligonucleotide primers, results of whole-cell biosensor absorbance, and fluorescence measurements (PDF)
