# Supplementary material for: Development and Application of Whole-Cell Biosensors for the Detection of Gallic Acid
Source: ACS Synth Biol. 2023 Feb 1;12(2):533–43. doi: 10.1021/acssynbio.2c00537 (PMC9942251; doi:10.1021/acssynbio.2c00537)
Supplement: Supplementary file 1 — sb2c00537_si_001.pdf [file sb2c00537_si_001.pdf]

Supporting Information

Development and application of whole-cell biosensor for detection of gallic acid

Ingrida Kutraite[1] and Naglis Malys[1,2]*

[1]Bioprocess Research Centre, Faculty of Chemical Technology, Kaunas University of Technology, Radvilėnų street 19, LT-50254 Kaunas, Lithuania

[2]Department of Organic Chemistry, Faculty of Chemical Technology, Kaunas University of Technology, Radvilėnų street 19, LT-50254 Kaunas, Lithuania

* Author to whom correspondence should be addressed; E-Mail: naglis.malys@ktu.lt

Supplementary Tables

Supplementary Table S1. Parameters of *E. coli* and *P. putida*-based whole-cell biosensors, cultivated in MM medium.

Whole-cell biosensor	Time, h	b_{max}	b_{min}	Dynamic range, -fold	K_m, mM	Hill coefficient
BS1	6 h	2241	5.698	246 ± 41	0.194 ± 0,04	1.17 ± 0.09
BS1	12 h	2985	11.24	263 ± 34	0.2 ± 0.01	1.34 ± 0.01
BS2	6 h	262.3	12.99	20 ± 1	0.233 ± 0.02	2.096 ± 0.09
BS2	12 h	527.8	4.32	124 ± 15	0.255 ± 0.01	2.203 ± 0.03

Data are mean ± SD, n = 3.

Supplementary Table S2. Chemicals used in this study.

Chemical	Supplier	Catalog number
Gallic acid hydrate	Fluorochem	242843
Sodium salicylate	Sigma-Aldrich	71945
Sodium 4-hydroxybenzoate	Fluorochem	047887
m-Hydroxybenzoic acid	Sigma-Aldrich	H20008
Vanillic acid	Sigma-Aldrich	94770
Isovanillic acid	Alfa Aesar	A13709
Protocatechuic acid	Alfa Aesar	B24016
Syringic acid	Alfa Aesar	A11725
Gentisic acid	Sigma-Aldrich	149357
α-Resorcylic acid	Sigma-Aldrich	D110000
β-Resorcylic acid	Sigma-Aldrich	D109401
γ-Resorcylic acid	Sigma-Aldrich	D109606
o-Orsellinic acid hydrate	Acros Organics	A0411384
6-Methylsalicylic acid	Acros Organics	341500
EGCG	Acros Organics	449010100
ECG	Fisher Scientific	15473519

Supplementary Table S3. Strains used in this study.

Strains and plasmids	Characteristics	Source/reference
Escherichia coli Top10	F^- *mcrA* Δ(*mrr-hsdRMS-mcrBC*) Φ80*lacZ*ΔM15 Δ*lacX*74 *recA*1 *araD*139 Δ(*araleu*)7697 *galU galK rpsL* (StrR) *endA1 nupG*	Thermo Fisher Scientific
Cupriavidus necator H16	Wild type strain	DSM 428
Pseudomonas putida KT2440	Wild type strain	DSM 291

Supplementary Table S4. Oligonucleotide primers used in this study. Restriction sites are underlined.

Primer name	Primer sequence (5' → 3')
EV001-PP_2515	gggcctttcgttttatgacgtctcacgcctgctcggtgat
EV001A	gggcctttcgttttatgacgtcaagggctcccggcgctcggctcgtga
EV001B	caggatggccttctgcttaacttatcacgcctgctcggtgat
EV001E	ctactttaaggcaccctcctg
EV003-PP_2515B	cgtcttcgctactcgccatatgtcgttggctcctgcagtgg
EV003C	gggcctttcgttttatgacgtcttagtttcgttggctcctgcagt
EV008-PP	ttaagcagaaggccatcctgacggatggcctttttgcgtttctacggaaggccctgtatcaatccc
EV008B	gggcctttcgttttatgacgtcggaaggccctgtatcaatccc
EV009-PP	cgtcttcgctactcgccatatgtggtcacctttgttcttgtattgg
IK023	aaggtgaccacgcatgcttgccgtaacctgtaacagaggactttcgc
IK024	caggagggtgccttaaagtagtttcattatttttattctccagggatgggcaa
IK025	aagcatgcgtggtcacctt
IK026	aaggtgaccacgcatgcttaactgaccgatgcaacccg
IK027	cgggttgcatcggtcagttagccatgcgaaagtcctctgttac
IK028	aactgaccgatgcaacccg
IK003	tcgttttatggcgcgccaggccggccaattagaaggccgccagagagg
IK004	actagtactgtttaaaccgctcacaattccacacaa

Supplementary Figures

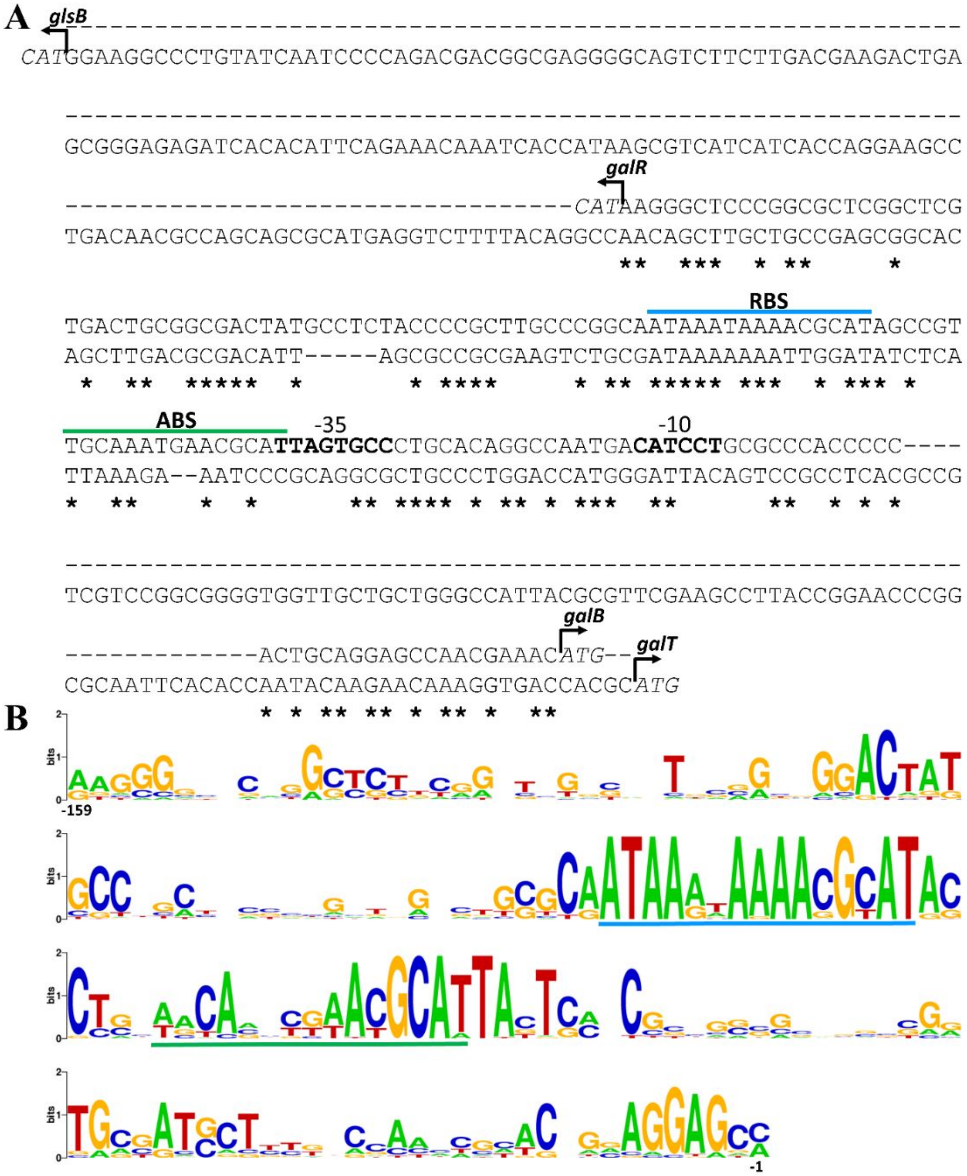

Supplementary Figure S1. Identification of regulatory elements in *P. putida* KT2440 *PP_RS13150*/*PP_RS13155* intergenic region. **(A)** The alignment of *P. putida* KT2440 *PP_RS13150*/*PP_RS13155* (*galB*/*galR*) and *PP_RS13170*/*PP_RS13175* (*galT*/*glsB*) intergenic regions. Translational start sites are italicized. The predicted *galB* −35 and −10 regions are bold. Identical nucleotides are indicated with asterisks. **(B)** A sequence similarity motif of *PP_RS13150*/*PP_RS13155* intergenic region of forty analyzed *Pseudomonas*. In **(A)** and **(B)** GalR regulatory binding site (RBS) is highlighted with blue line, whereas the activation binding site (ABS) is in green.

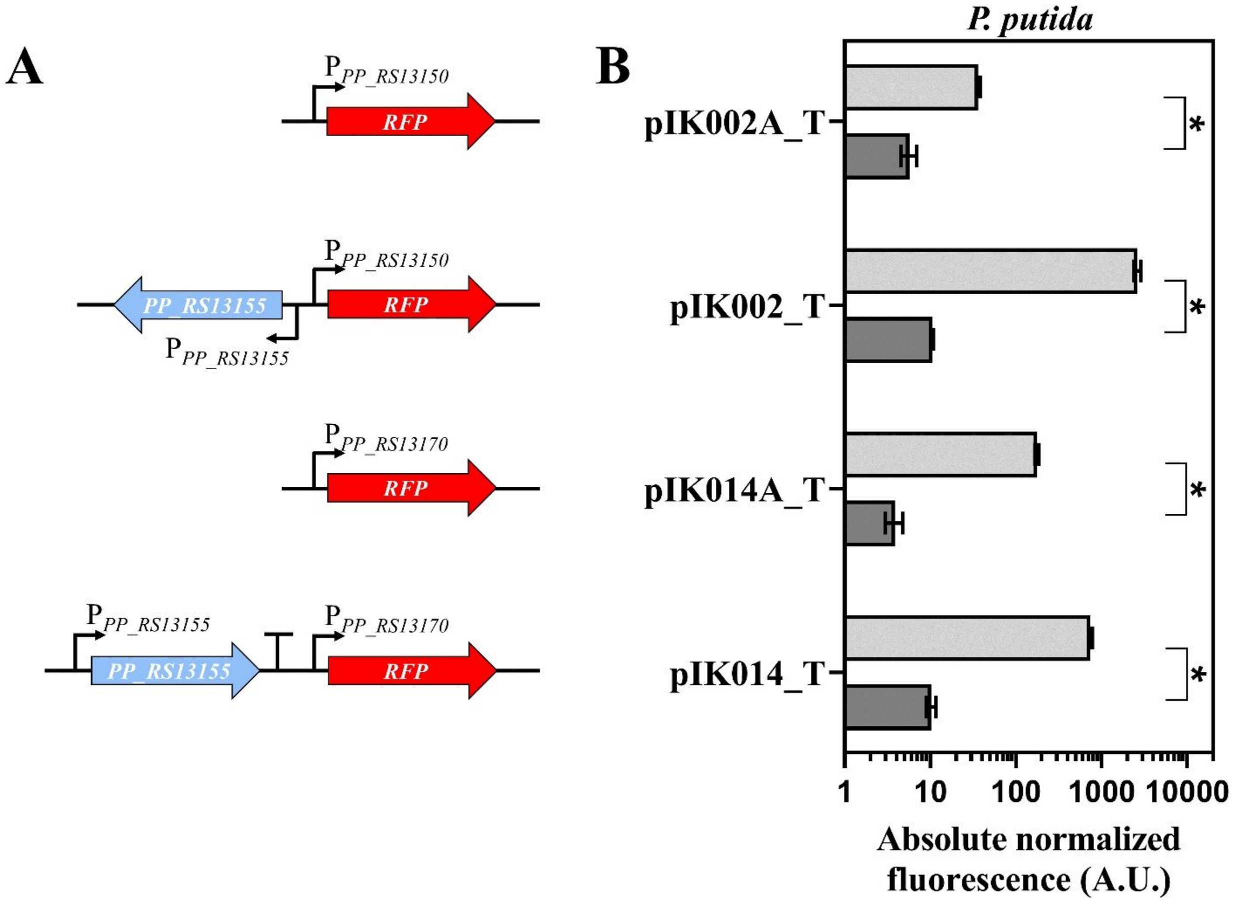

Supplementary Figure S2. Evaluation of inducible gene expression systems responding to exogenous gallic acid in *P. putida* and analysis of genetic elements required for gene expression activation. Comparison of absolute normalized fluorescence **(B)** of *P. putida* cells harboring inducible systems assembled in constructs pIK002A_T, pIK002_T, pIK014A_T, and pIK014_T **(A)**. The absolute normalized fluorescence was determined using absorbance and fluorescence values measured 6 hours after exogenous addition of gallic acid to the logarithmically growing cells in MM medium at the final concentration of 1.25 mM (light grey) or 0 mM (dark grey). Data represent mean values ± standard deviations (SD) of three biological replicates, $*p<0.001$ (unpaired *t*-test).

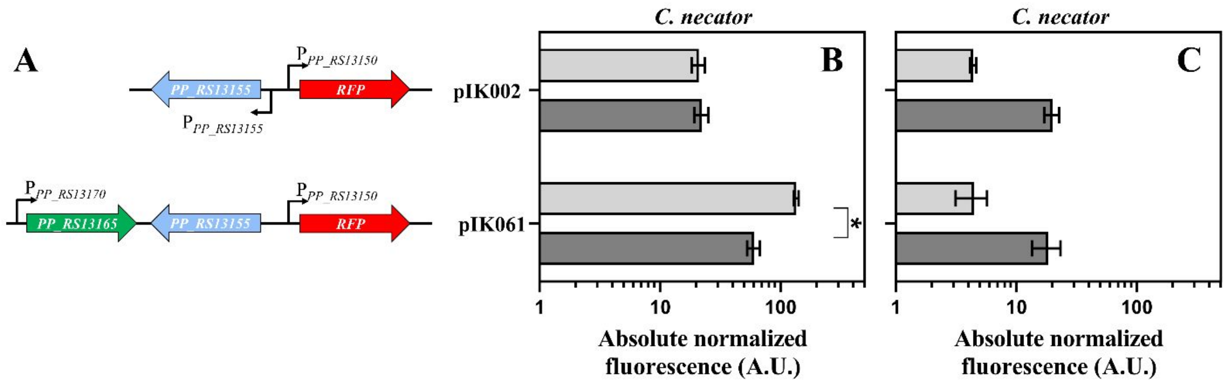

Supplementary Figure S3. Evaluation of gallic acid biosensor based on non-native host *C. necator* H16. **(A)** The genetic organization of inducible system's variants supplemented with different sets of genes. Results represent absolute normalized fluorescence in LB **(B)**, and MM medium **(C)** 6 hours after exogenous addition of gallic acid to the final concentration of 5 mM (light grey) or 0 mM (dark grey), Data represent mean values ± SD of three biological replicates, *p<0.001(unpaired *t*-test).

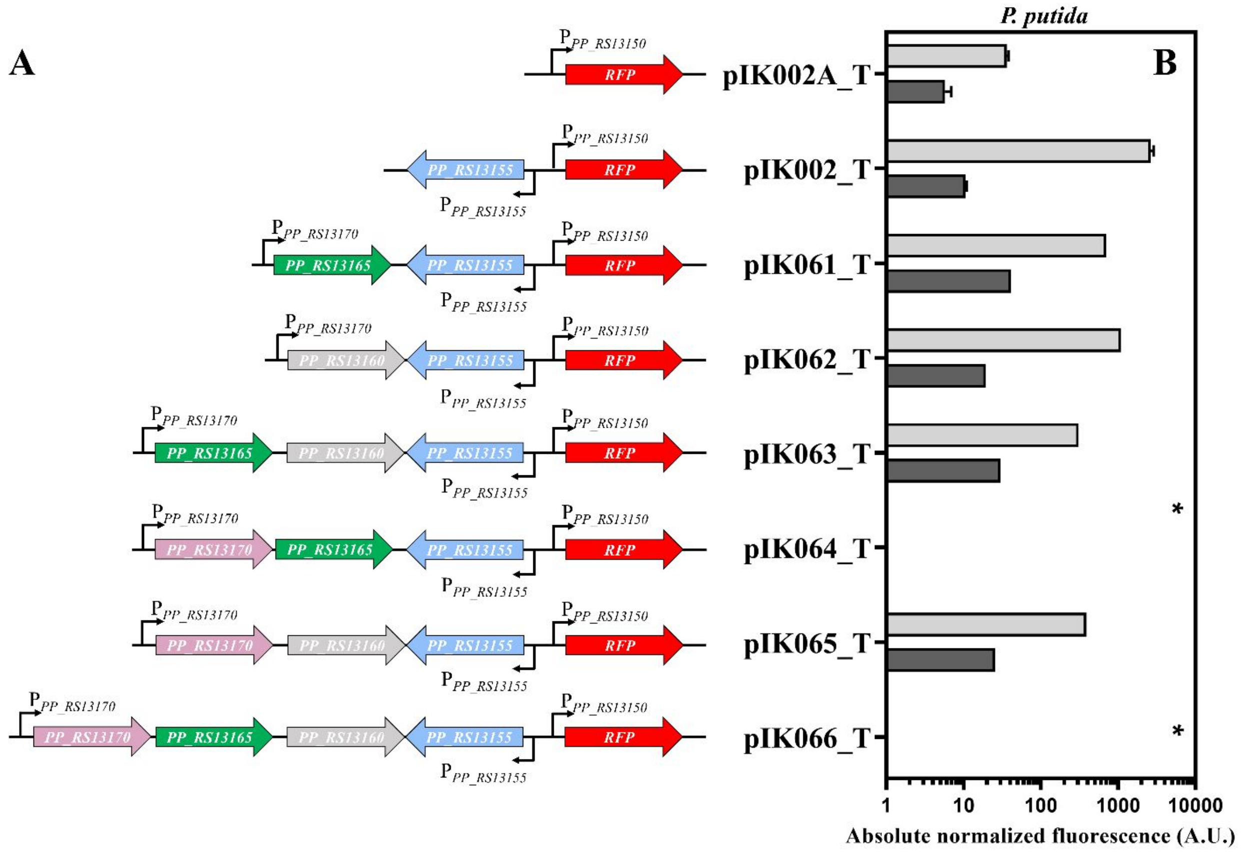

Supplementary Figure S4. Results of absolute normalized fluorescence using *P. putida* KT2440 as a host of application 6 hours after exogenous addition of gallic acid to the final concentration of 5 mM (light grey) or 0 mM (dark grey) in MM medium. Data represent values of one biological replicate, excluding pIK002A_T and pIK002_T, representing mean values ± SD of three biological replicates. Asterisks indicate those cases where stable transformation was not possible to achieve.

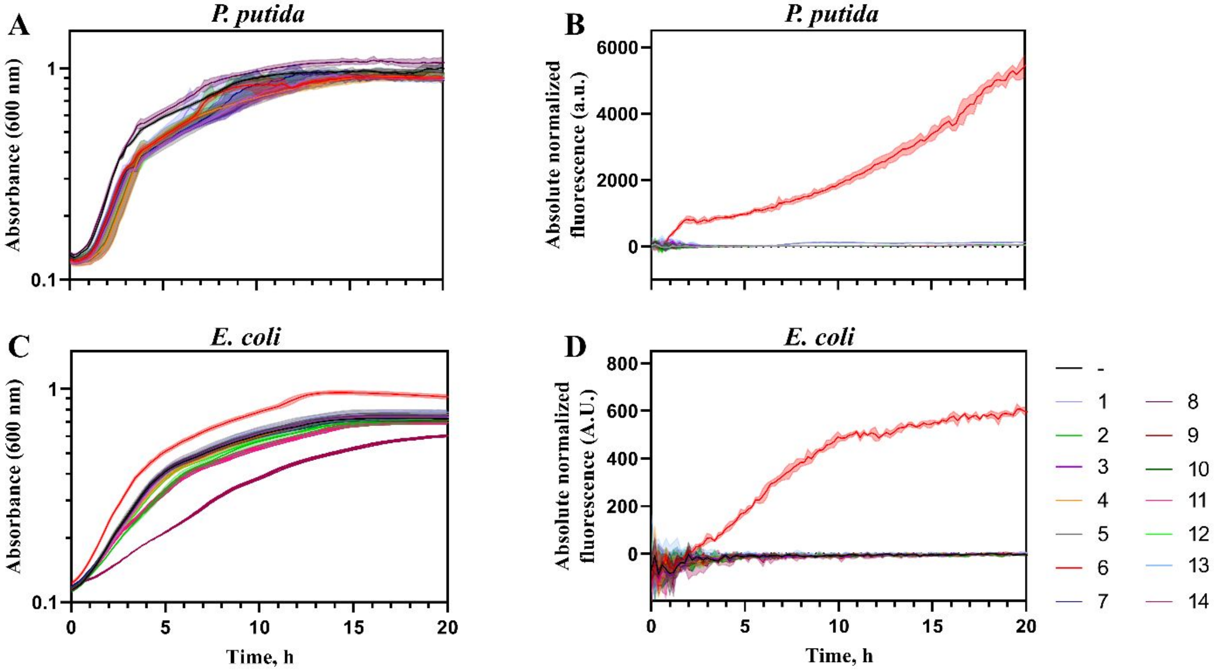

Supplementary Figure S5. The analysis of specificity of *P. putida*- and *E. coli*-based biosensors BS1 (*Pp*GalR/P$_{PP_RS13150}$) and BS2 (*Pp*GalRGalAGalT/P$_{PP_RS13150}$) towards different phenolic acids. Absorbance of BS1 **(A)** and BS2 **(C)** and absolute normalized fluorescence of BS1 **(B)** and BS2 **(D)** were measured every 10 min for 20 hours after following phenolic acids were added to the cell culture at the final concentration of 1.25 mM: *p*-hydroxybenzoic acid (1), salicylic acid (2), *m*-hydroxybenzoic acid (3), vanillic acid (4), isovanillic acid (5), gallic acid (6), protocatechuic acid (7), syringic acid (8), gentisic acid (9), *α*-resorcylic acid (10), *β*-resorcylic acid (11), *γ*-resorcylic acid (12), orsellinic acid (13), 6-methylsalicylic acid (14). Data represent mean values with error bars corresponding to standard deviations of three biological replicates.

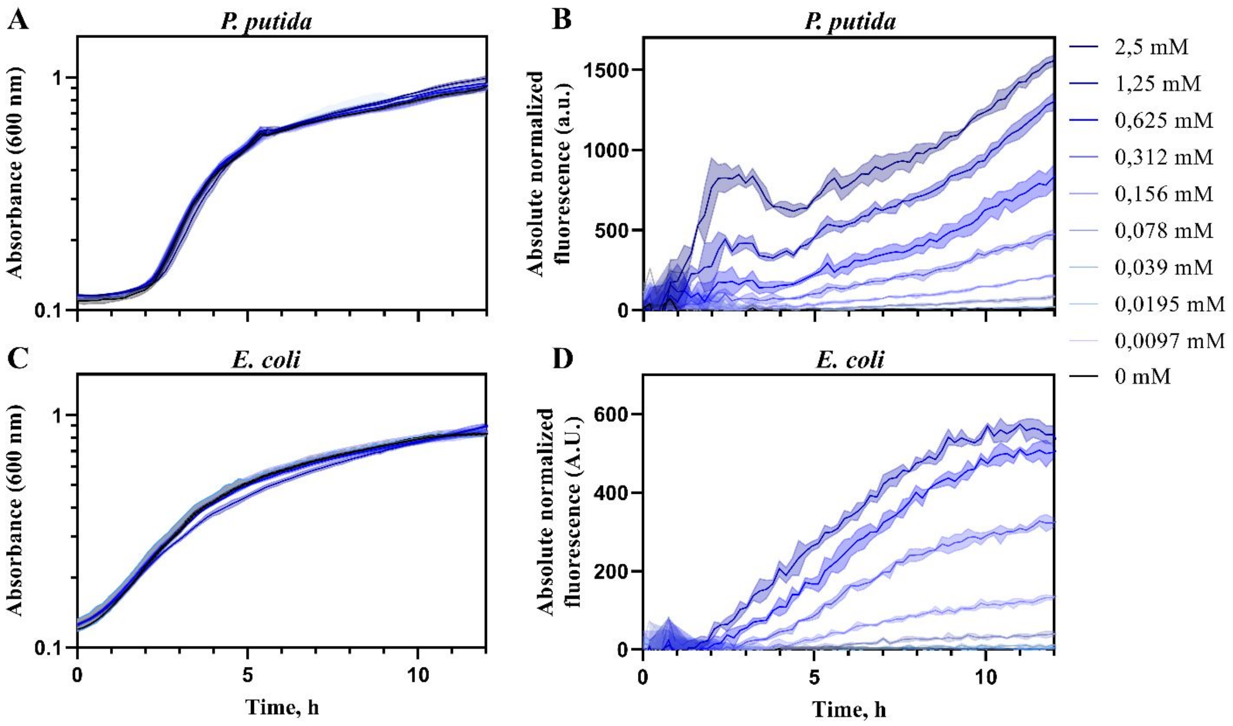

Supplementary Figure S6. The sensitivity of biosensors to gallic acid of concentrations ranging from 0 to 2.5 mM, measured in LB medium. Absorbance at 600 nm of *P.putida*/*Pp*GalR/$P_{PP\_RS13150}$ (BS1) **(A)** and *E.coli*/*Pp*GalR,GalA,GalT/$P_{PP\_RS13150}$ (BS2) **(C)** biosensors. Absolute normalized fluorescence of BS1 **(B)** and BS2 **(D)**, using 585 nm as excitation wavelength and 620 nm as emission wavelength. Data represent mean values with error bars corresponding to standard deviations of three biological replicates.

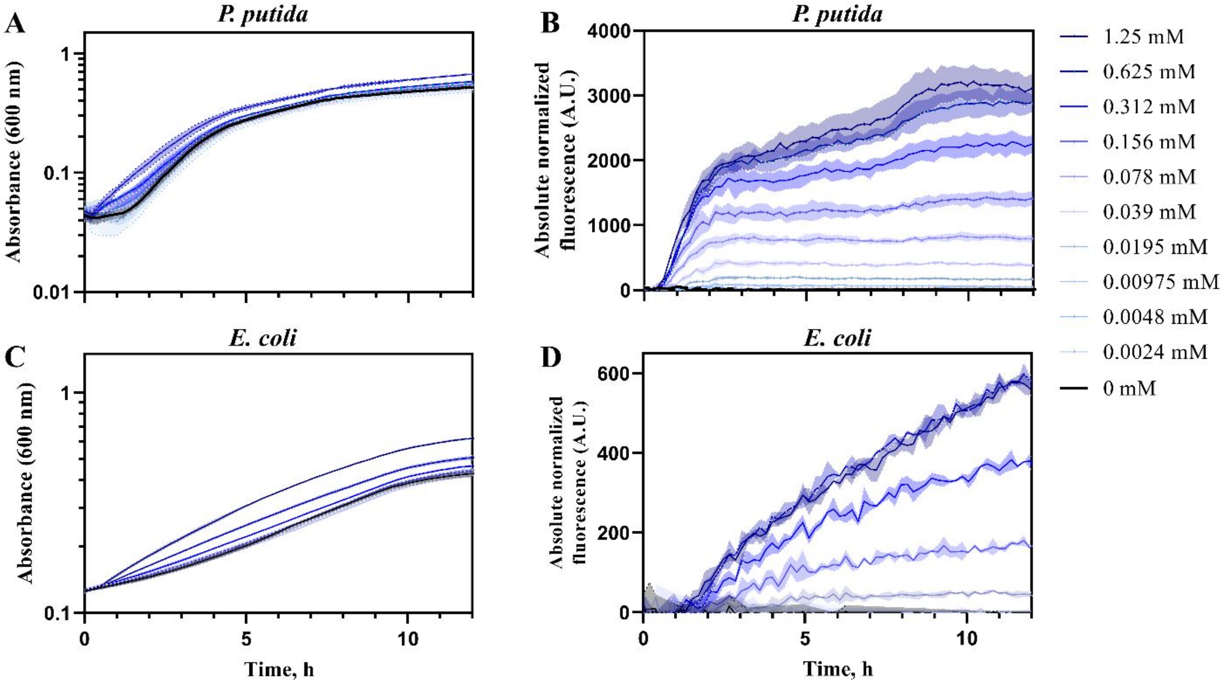

Supplementary Figure S7. The sensitivity of biosensors to gallic acid of concentrations ranging from 0 to 1.25 mM, measured in MM medium. Absorbance at 600 nm of *P.putida*/*Pp*GalR/$P_{PP\_RS13150}$ (BS1) **(A)** and *E.coli*/*Pp*GalR,GalA,GalT/$P_{PP\_RS13150}$ (BS2) **(C)** biosensors. Absolute normalized fluorescence of BS1 **(B)** and BS2 **(D)**, using 585 nm as excitation wavelength and 620 nm as emission wavelength. Data represent mean values and error bars corresponding to standard deviations of three biological replicates.

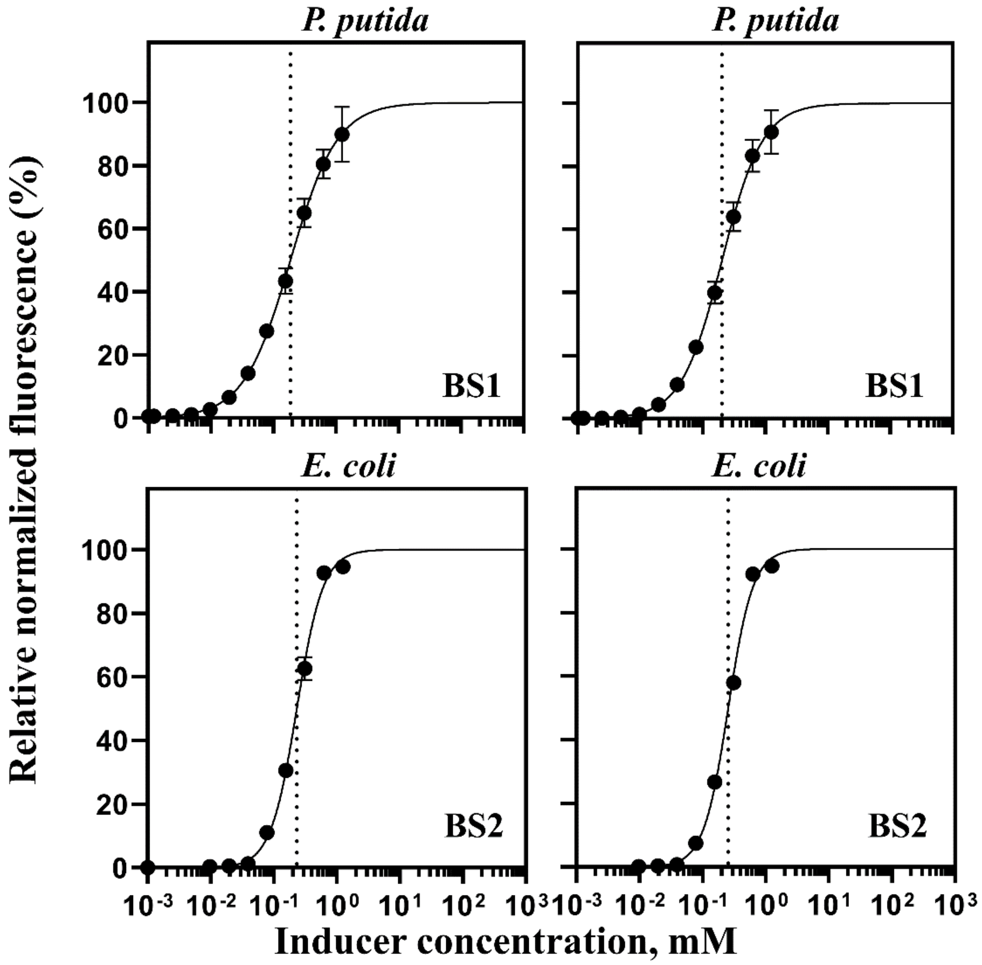

Supplementary Figure S8. Dose-response curves of gallic acid-inducible biosensors. Relative normalized fluorescence of BS1 and BS2 6 hours (left) and 12 hours (right) after addition of different concentrations of gallic acid, ranging from 0 to 1.25 mM in MM medium. The dose-response curves were fitted using the Hill function as described in *Methods*. K_m is indicated by a dotted line. Data represent mean values ± SD of three biological replicates.

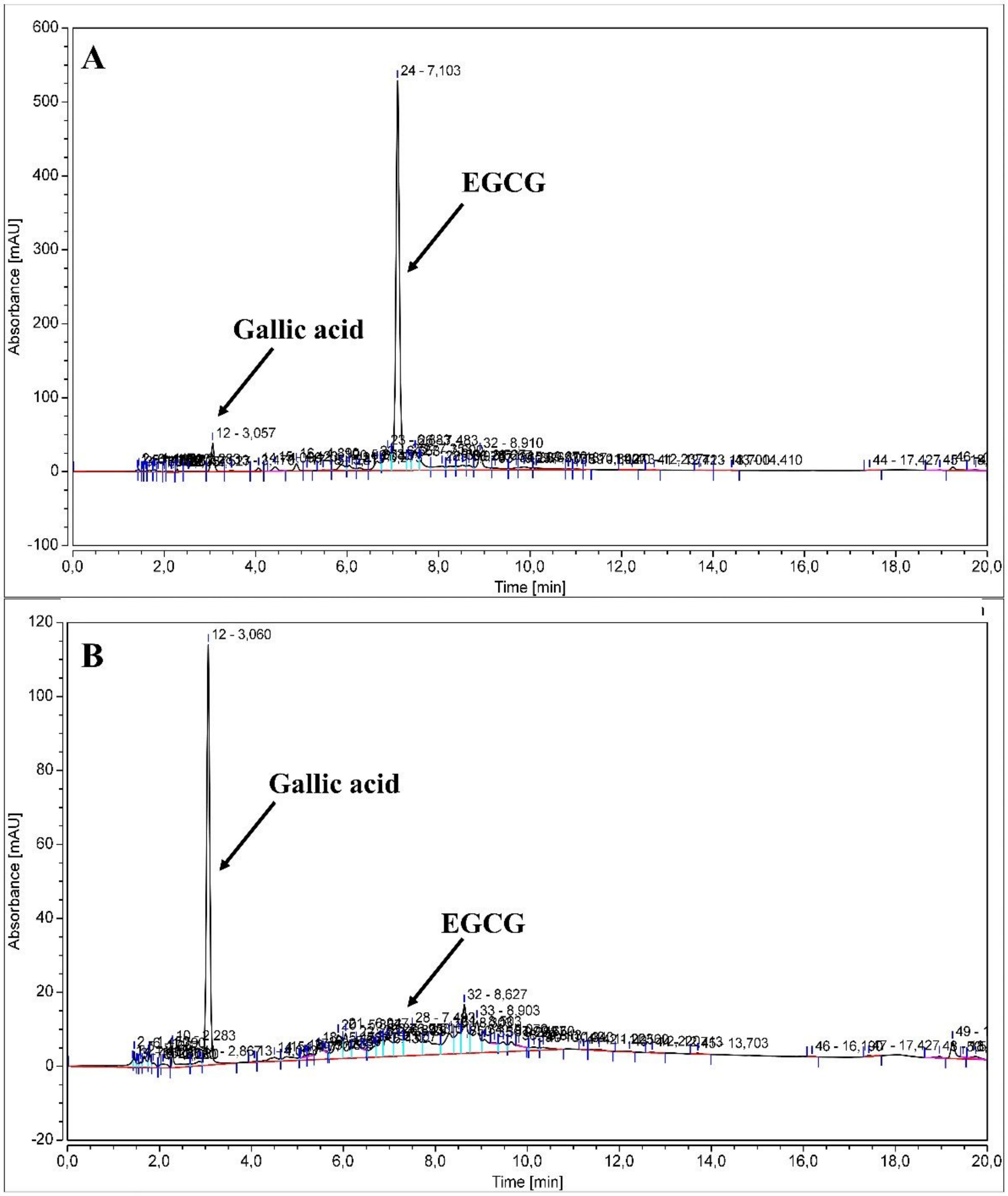

Supplementary Figure S9. HPLC results for EGCG stability in MM medium. **(A)** EGCG standard at 0 h, **(B)** EGCG standard after 24 h.

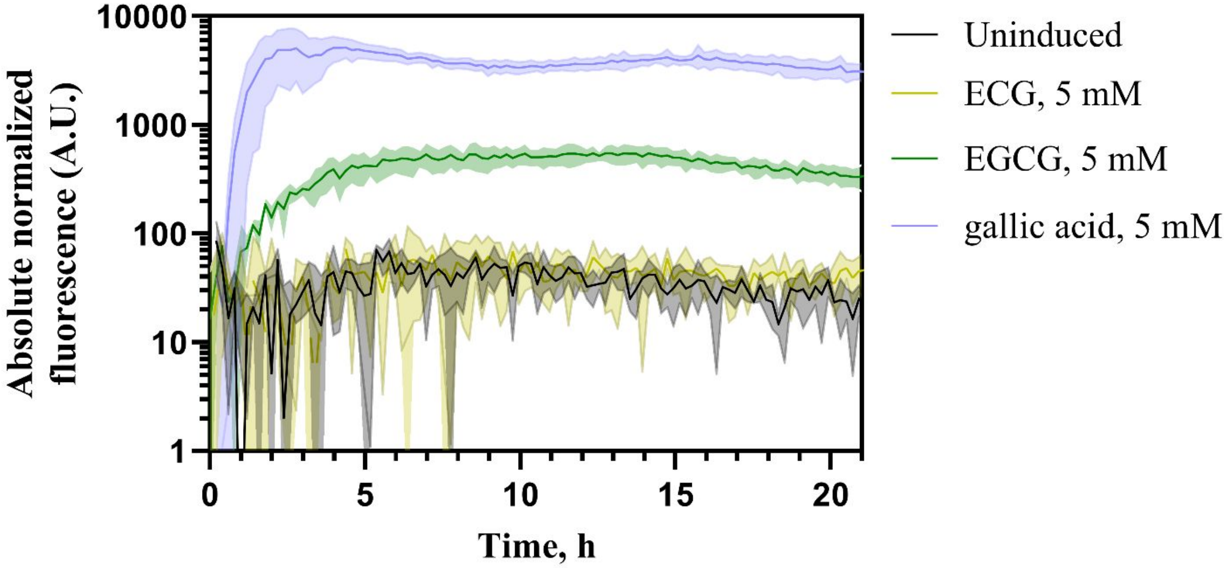

Supplementary Figure S10. Absolute normalized fluorescence of BS1 cultivated in MM medium with 5 mM EGCG, 5 mM ECG, and 5 mM gallic acid, using 585 nm as excitation wavelength and 620 nm as emission wavelength. Data represent mean values with error bars corresponding to standard deviations of three biological replicates.

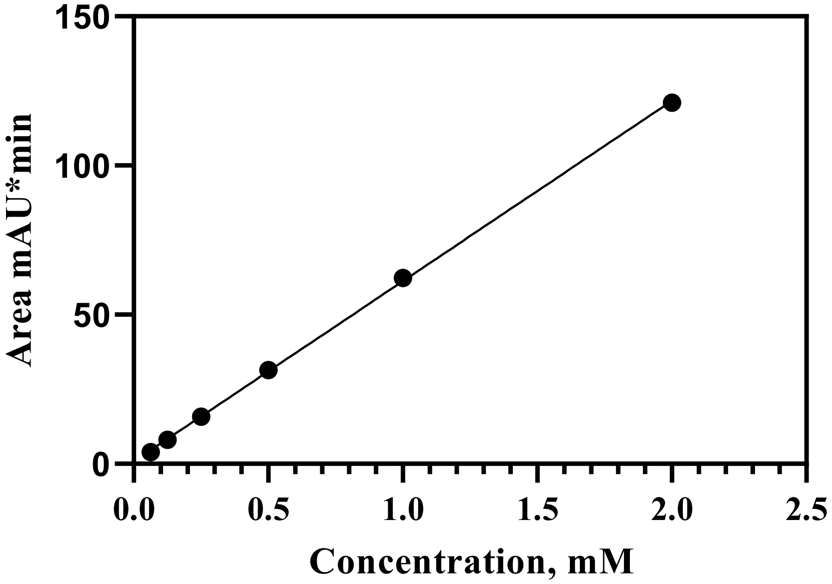

Supplementary Figure S11. HPLC calibration curve of gallic acid standards of concentrations ranging from 0.0625 to 2 mM error bars correspond to standard deviations of three biological replicates. The received equation Y = 60,46*X + 0,7670.
